# Genome-wide identification and expression analysis of fibrillin (*FBN*) gene family in tomato (*Solanum lycopersicum* L.)

**DOI:** 10.7717/peerj.13414

**Published:** 2022-05-09

**Authors:** Huiru Sun, Min Ren, Jianing Zhang

**Affiliations:** 1 College of Life Sciences, Yan’an University, Yan’an, Shaanxi Province, China; 2 Shaanxi Key Laboratory of Chinese Jujube, Yan’an University, Yan’an, Shaanxi Province, China

**Keywords:** Tomato, FBN genes family, Expression analysis, Leaf, Fruit

## Abstract

**Background:**

Fibrillin (FBN) proteins are widely distributed in the photosynthetic organs. The members of *FBN* gene family play important roles in plant growth and development, and response to hormone and stresses. Tomato is a vegetable crop with significantly economic value and model plant commonly used in research. However, the FBN family has not been systematical studied in tomato.

**Methods:**

In this study, 14 *FBN* genes were identified in tomato genome by Pfam and Hmmer 3.0 software. ExPASy, MEGA 6.0, MEME, GSDS, TBtools, PlantCARE and so on were used for physical and chemical properties analysis, phylogenetic analysis, gene structure and conserved motifs analysis, collinearity analysis and cis-acting element analysis of *FBN* family genes in tomato. Expression characteristics of *SlFBNs* in different tissues, fruit shape near isogenic lines (NILs), *Pst* DC3000 and ABA treatments were analyzed based on transcriptome data and quantitative Real-time qPCR (qRT-PCR) analysis.

**Results:**

The *SlFBN* family was divided into 11 subgroups. There were 8 *FBN* homologous gene pairs between tomato and *Arabidopsis*. All the members of SlFBN family contained PAP conserved domain, but their gene structure and conserved motifs showed apparent differences. The cis-acting elements of light and hormone (especially ethylene, methyl jasmonate (MeJA) and abscisic acid (ABA)) were widely distributed in the *SlFBN* promoter regions. The expression analysis found that most of *SlFBNs* were predominantly expressed in leaves of Heinz and *S. pimpinellifolium* LA1589, and showed higher expressions in mature or senescent leaves than in young leaves. Expression analysis of different tissues and fruit shape NILs indicated *SlFBN1*, *SlFBN2b* and *SlFBN7a* might play important roles during tomato fruit differentiation. All of the *SlFBNs* responded to *Pst* DC3000 and ABA treatments. The results of this study contribute to exploring the functions and molecular mechanisms of *SlFBNs* in leaf development, fruit differentiation, stress and hormone responses.

## Introduction

Plastoglobules (PGs) are lipoprotein particles in plastid, which are involved in plant growth and development, and stress resistance. Fibrillin (FBN) proteins are plastid lipid-associated and highly conserved, which encoded by nuclear genes and are the most abundant in chloroplast PGs (Singh & McNellis, 2011). FBNs showed important regulatory roles in plastid stability, plant growth and development, and stress response (Rey et al., 2010; Simkin et al., 2007; Youssef et al., 2010; Ytterberg, Peltier & van Wijk, 2006).

FBN proteins contain a conserved plastid-lipids associated protein (PAP) domain and are widely exist in photosynthetic organisms from cyanobacteria to plants (Cunningham et al., 2010; Lohscheider & Bártulos, 2016; Simkin et al., 2007). FBN proteins were early found in the chromoplasts of red rose (*Rosa rugosa*) (Wuttke, 1976) and bell pepper (*Capsicum annuum*) fruit (Deruère et al., 1994). Subsequently, the similar proteins were isolated from thylakoids of potato (*Solanum tuberosum*) leaves (Pruvot et al., 1996), chromoplast in cucumber (*Cucumis sativus*) petals (Vishnevetsky et al., 1996), chloroplast PG of *Arabidopsis thaliana* leaves (Vidi et al., 2006) etc, which have been named PGL, PAP and FBN. Ultimately, these similar proteins are collectively referred to as FBN (Singh & McNellis, 2011). So far, the FBN families in different species have been divided into 12 groups (FBN1–FBN12) (Kim et al., 2018). The FBN12 group is only present in lower algal fungi (Lohscheider & Bártulos, 2016). Besides the unique PAP domain, FBN11 group also contain a protein kinase C domain (PKC), which indicates that members of this group might have other functions that need to be studied other than lipoprotein-related functions (Li et al., 2020). Moreover, the isoelectric point and molecular weight ranges of FBN family are wide, and they are distributed in different plastids, including chloroplasts, elaioplasts, chromoplasts and etioplasts, which might be related to the diversified functions (Kim et al., 2018).

The *FBN* family genes have showed important roles in various processes, such as plastid structure stabilization, organ development, stress response and hormone signal transmission (Kim, Lee & Kim, 2015). FBN1 protein was detected in the chromoplasts of ripe pepper fruits, and overexpression of this gene promoted the increase and aggregation of PGs in chromoplasts (Jotham et al., 2006). In addition, GUS activity of *FBN1* promoter increased with tomato (*Solanum lycopersicum*) leaf aging (Georg et al., 2001). The expression of *Pap2* (*FBN1b*) in *Brassica rapa* decreases with the aging of leaves (Kim, Wu & Huang, 2001). The content of FBN1 protein in bell pepper gradually increased with fruit ripening and reached the peak at the full fruit ripening stage. *C40.4* (*FBN1*) in potato was expressed in leaves and multiple flower organs, and inhibition of this gene expression leaded to plant growth retardation and smaller tuber size (Monte, Ludevid & Prat, 2010). Some *FBN* family genes in rice (*Oryza sativa*) were respond to ABA and extreme temperature treatments (Lee et al., 2007; Li et al., 2020). *CsaFBN1*, *CsaFBN6* and *CsaFBN11* in cucumber were induced and up-regulated expressed under high-light and low temperature stress (Kim et al., 2018). The RNAi of *LeChrC* (*FBN1*) and mutants of *fbi4* in apple (*Malus domestica*) and *Arabidopsis* were more sensitive to *Botrytis cinerea, Erwinia amylovora* and *Pseudomonas syringae*, respectively (Leitner-Dagan et al., 2006; Singh et al., 2010). The *fbn6* mutant of *Arabidopsis* showed resistance to cadmium and high-light stresses (Lee et al., 2020). The expression of jasmonic acid (JA) synthesis related genes in *fbn5* mutants were inhibited under high-light stress (Otsubo et al., 2018). Under drought stress, *FBN1* showed significantly decreased expression in tomato *flacca* mutant with deficient in ABA synthesis (Gillet et al., 1998). *FBN1* and *FBN2* responded to light and cold stresses through JA synthesis pathway (Youssef et al., 2010). Some *FBN* family members in wheat (*Triticum aestivum*) were involved in the response to drought, cold, heat and stripe rust stresses (Jiang et al., 2020).

Tomato has rich nutritional value and is widely cultivated in the world as an important vegetable crop (Albacete et al., 2008). According to the Food and Agriculture Organization of the United Nations (FAO) statistics, the tomato cultivated area in the world has exceeded five million hectares, and the production has reached 187 million tons in 2020 (http://www.fao.org/faostat/en/#data/QC). The *FBN* family genes play key regulatory roles in many biological processes such as plant development and stress response. However, the vast majority of *FBN* family genes in tomato are unknown, and no comprehensive analysis of this family in tomato has been reported. In this study, bioinformatics methods were used to identify and analyze the phylogenetic relationship, conserved domain, collinearity, cis-acting element in promoter regions of *SlFBN* gene family. The expression characteristics of *SlFBNs* in different tissues, *Pst* DC3000 and ABA treatments were analyzed using transcriptome data and qRT-PCR, which provided a theoretical foundation for exploring the potential functions of *SlFBNs*.

## Materials and Methods

### Tomato material and treatments

The tomato used in this study was Micro-Tom. The tomato seedings grew under normal temperature conditions (26 °C/16 h in light condition and 18 °C/8 h in dark condition). When the seedings grew to 6-leaf stage, the young leaves, mature leaves and aging leaves (the yellowing part less than 5% of the whole leaf) were collected. The 6-leaf seedings with similar growth were treated with 100 μM ABA, and water as a control group. The leaves were collected at 0, 6, 12 and 24 h after treatments. The samples were immediately frozen in liquid nitrogen and stored at −80 °C. All samples were tested with tree independent biological replicates.

### Identification of FBNs in tomato

Genomic data of tomato was downloaded from Sol Genomics Network (SL4.0, http://solgenomics.net/). The genomes of *Arabidopsis*, rice, maize (*Zea mays*), sorghum (*Sorghum bicolor*), cucumber and pepper were downloaded from TAIR (http://www.arabidopsis.org/), phytozome (https://phytozome.net/) and PGP (https://db.cngb.org/search/assembly/GCF_000710875.1/) databases, respectively. The PAP domain model of FBN family was obtained from Pfam database (http://pfam.xfam.org). *FBN* genes in tomato genome were screened by HMMER 3.0 based on the model (the threshold set as *E* < 1e−4). The normal mode of SMART (http://smart.embl-heidelberg.de/) and CD–search (https://www.ncbi.nlm.nih.gov/Structure/cdd/wrpsb.cgi) were used to further verify *SlFBN* family genes. Finally, members of *FBN* family in tomato were determined. The lengths, molecular weights(Mws), isoelectric points (pIs) and hydrophilicities of SlFBN proteins were predicted by ExPASy website (http://web.expasy.org/protparam/) (Artimo et al., 2012). The subcellular localizations of tomato FBN proteins were predicated using WoLF PSORT (https://wolfpsort.hgc.jp/) (Paul et al., 2007).

### Phylogenetic tree, gene structure and conserved domain analysis

The FBN protein sequences from tomato, *Arabidopsis*, rice, maize, sorghum, cucumber and pepper were aligned and constructed phylogenetic tree using MEGA 6.0 by the neighbor-joining (NJ) with the p-distance model, and bootstrap was set to 1,000 replications.

The gene structures of *SlFBNs* were drawn in GSDS 2.0 (http://gsds.cbi.pku.edu.cn/) based on the introns-exon position information (Hu et al., 2015). The PAP domains of SlFBN proteins were analyzed using SMART website. The conserved motifs of SlFBN proteins were identified by MEME website (https://meme-suite.org/meme/). The maximum number of motif findings was set to 10, and other parameters were set to default values (Bailey et al., 2009).

### Chromosome location and collinearity analysis

The chromosome distribution of *SlFBNs* was drawn by TBtools according to the location information from tomato genome database (Chen et al., 2020). MCScanx software was used to analyze the collinearity of *FBN* genes among *Arabidopsis*, rice and tomato (Wang et al., 2012), and then the collinearity diagram was shown through the Basic Circos module of TBtools.

### Cis-acting elements in FBN promoter regions

The 1.5 kb sequences located upstream of the start codon of tomato *FBN* family genes were extracted to analyze the cis-acting elements on the PlantCARE website (http://bioinformatics.psb.ugent.be/webtools/plantcare/html/) (Lescot et al., 2002).

### Transcriptome analysis of *SlFBNs* in different tissues and under *Pst* DC3000 treatment

The transcriptome data of *SlFBNs* in different tissues of Heinz and *S. pimpinellifolium* LA1589, under *Pst* DC3000 treatment in tomato varieties with different resistances (RG-PtoR: resistant, RG-prf3: sensitive and RG-prf9: sensitive) and in flower meristems at different developmental stages of LA1589 and three fruit shape near isogenic lines (NILs) in LA1589 background (WT, *sun*, *ovate* and *fs8.1*) (Wang et al., 2019) were obtained from TFGD (http://ted.bti.cornell.edu/cgi-bin/TFGD/digital/home.cgi). The expression heat maps of *SlFBNs* were drawn using TBtools.

### RNA extraction and quantitative real-time PCR (qRT-PCR)

Total RNA was extracted from the collected samples using the Total RNA Kit (TIANGEN, Beijing, China). The cDNA was synthesized from 1 μg using the StarScript II First-strand cDNA Synthesis Mix kit (GenStar, Beijing, China) according to the manufacturer’s instructions. Then qRT-PCR was carried out with an Applied Biosystems StepOnePlus using RealStar Green Fast Mixture with ROX (2×) (GenStar, Beijing, China). The gene-specific primers used for qRT-PCR were designed by Primer 5.0 (Table S1). The qRT-PCR was performed as follows: step 1: 95 °C for 2 min; step 2: 40 cycles of 95 °C for 15 s, 60 °C for 30 s; and step 3: melting curve analysis. Three biological replicates and the 2^−ΔΔCT^ method were used to calculate the relative expression level (Livak & Schmittgen, 2001). The tomato *ACTIN* gene (Solyc04g011500.3.1) was used as internal reference gene (Liu et al., 2022). The *t*-test was used to analyze the significance of the difference.

## Results

### Identification of *FBN* members in tomato

In our study, a total of 14 putative *FBN* genes were identified in tomato genome through screening using PAP domain (PF04755) and validating in SMART database. According to the homology with *FBNs* in *Arabidopsis*, *SlFBNs* were named as *SlFBN1–SlFBN11* (Table S2). The physical and chemical properties of SlFBN proteins were analyzed. The result showed that the lengths of SlFBN proteins ranged from 206 amino acids (aa) (SlFBN9) to 541 aa (SlFBN11). The predicated Mw and pI ranged from 23.43 kDa (SlFBN9)–61.35 kDa (SlFBN11), and 4.65 (SlFBN2b)–9.72 (SlFBN3a). The hydrophobicities of SlFBNs were less than zero, indicating that they were all hydrophilic proteins. Most (11/14) of SlFBNs were located in chloroplasts, except for SlFBN3 (nucleus), SlFBN11 (nucleus) and SlFBN9 (mitochondria) by subcellar location predication.

### Phylogenetic analysis of SlFBNs

**Figure 1:**
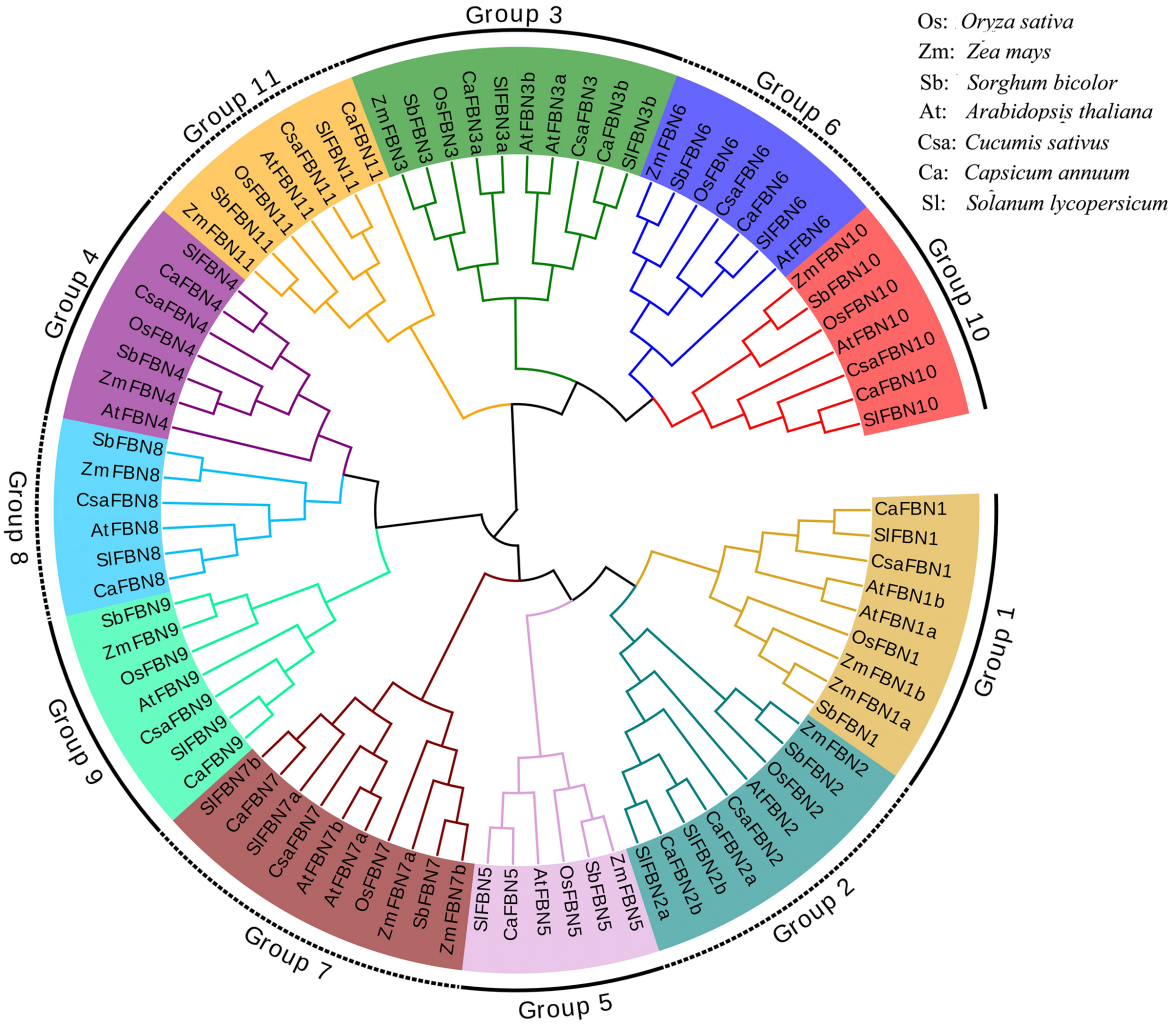
Phylogenetic tree of FBN family.

To detect the evolutionary relationships of SlFBN family, total 85 FBN proteins were collected from rice (10), maize (13), sorghum (11), *Arabidopsis* (14), cucumber (10), pepper (13) and tomato (14) to construct the phylogenetic tree (Fig. 1). These FBN proteins were divided into 11 groups (Group 1–Group 11) which distributed FBN members from mono-and dicotyledons suggesting that the differences of FBN groups were completed before the separation of mono-and dicotyledons. In some species, group 1, 2, 3 and 7 were amplified. FBN members of tomato and pepper, which belong to Solanaceae, were firstly cluster in the same branch, while the FBN members from mono- and dicotyledons were clustered distantly. These results suggested that FBN family members in mono- and dicotyledons showed different evolutionary characteristics.

### Chromosomal location and collinearity analysis of *FBN* genes

**Figure 2:**
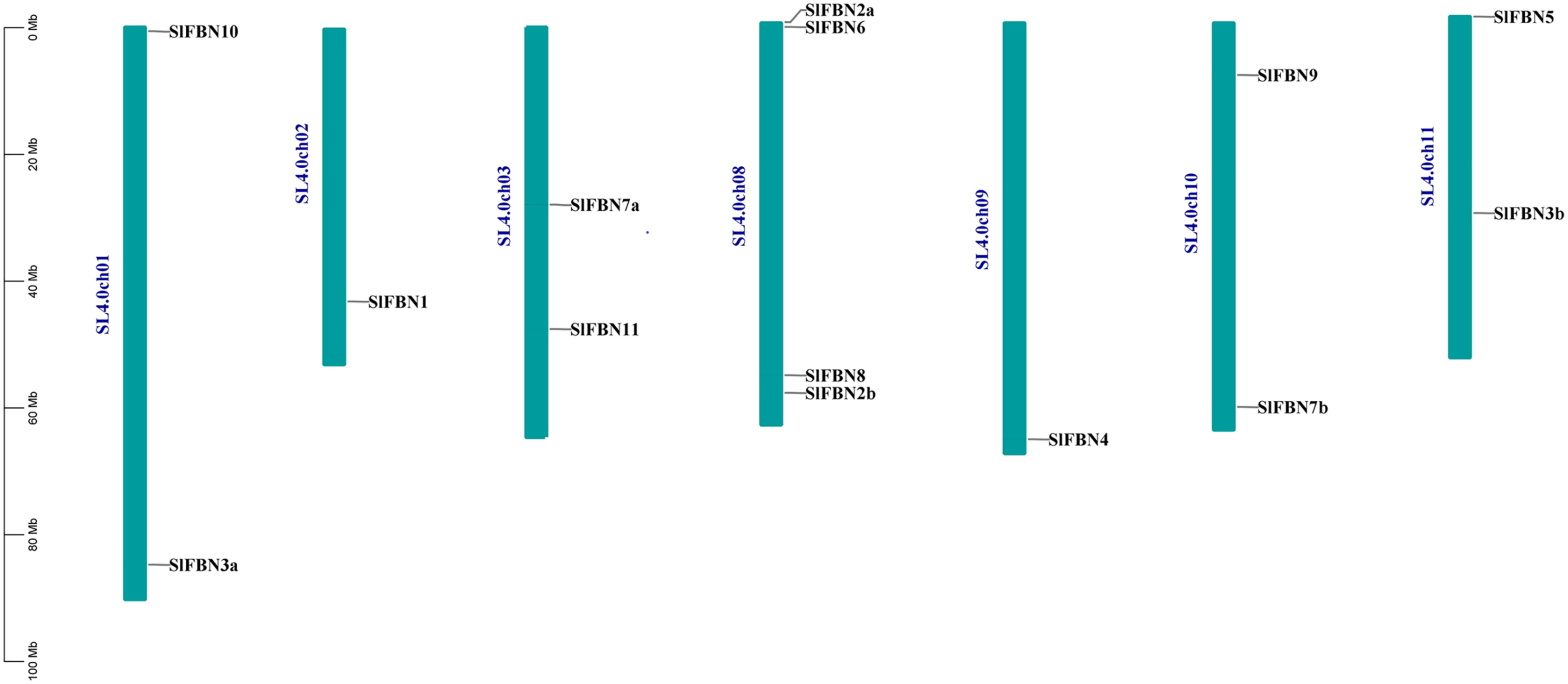
Chromosome location of *SlFBN* family genes.

**Figure 3:**
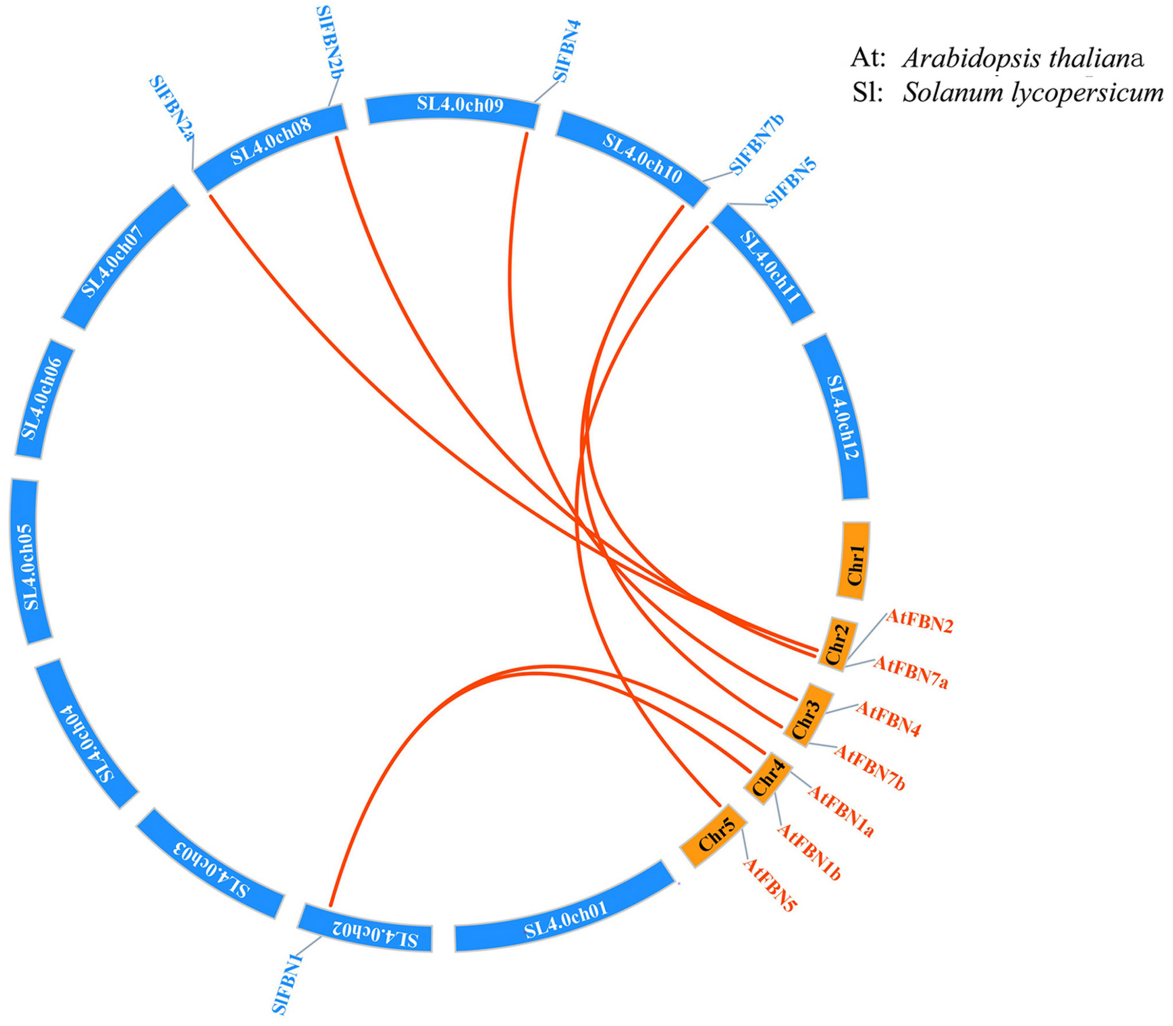
Collinear analysis of *FBNs* in tomato and *Arabidopsis thaliana*. The orange lines highlight the syntenic *FBN* gene pairs.

According to analysis of the chromosome positions, the 14 tomato *FBN* genes were unevenly distributed on seven chromosomes (Chr.01, Chr.02, Chr.03, Chr.08, Chr.09, Chr.10 and Chr.11). Among them, four *SlFBNs* were located on Chr.08, and 1 to 2 *SlFBNs* were located on the other six chromosomes (Fig. 2). The analysis of gene duplication event showed that there was no *FBN* gene duplication in tomato. To understand the collinearity relationship of *FBN* genes, the *FBN* homologous gene pairs between tomato and other plant species (*Arabidopsis* and rice) were found. There were eight collinear gene pairs between six *SlFBNs* and seven *AtFBNs*. *SlFBN1* and *SlFBN7b* were collinear with two *AtFBN* genes (*AtFBN 1a–AtFBN1b* and *AtFBN 7a–AtFBN7b*), respectively. *AtFBN2* was collinear with two *SlFBN* genes (*SlFBN2a–SlFBN2b*). *SlFBN4* and *SlFBN5* were collinear with one *AtFBN* gene, respectively (Fig. 3 and Table S3). There were no *FBN* homologous gene pairs between tomato and rice.

### Gene structure and conserved domain of SlFBNs

**Figure 4:**
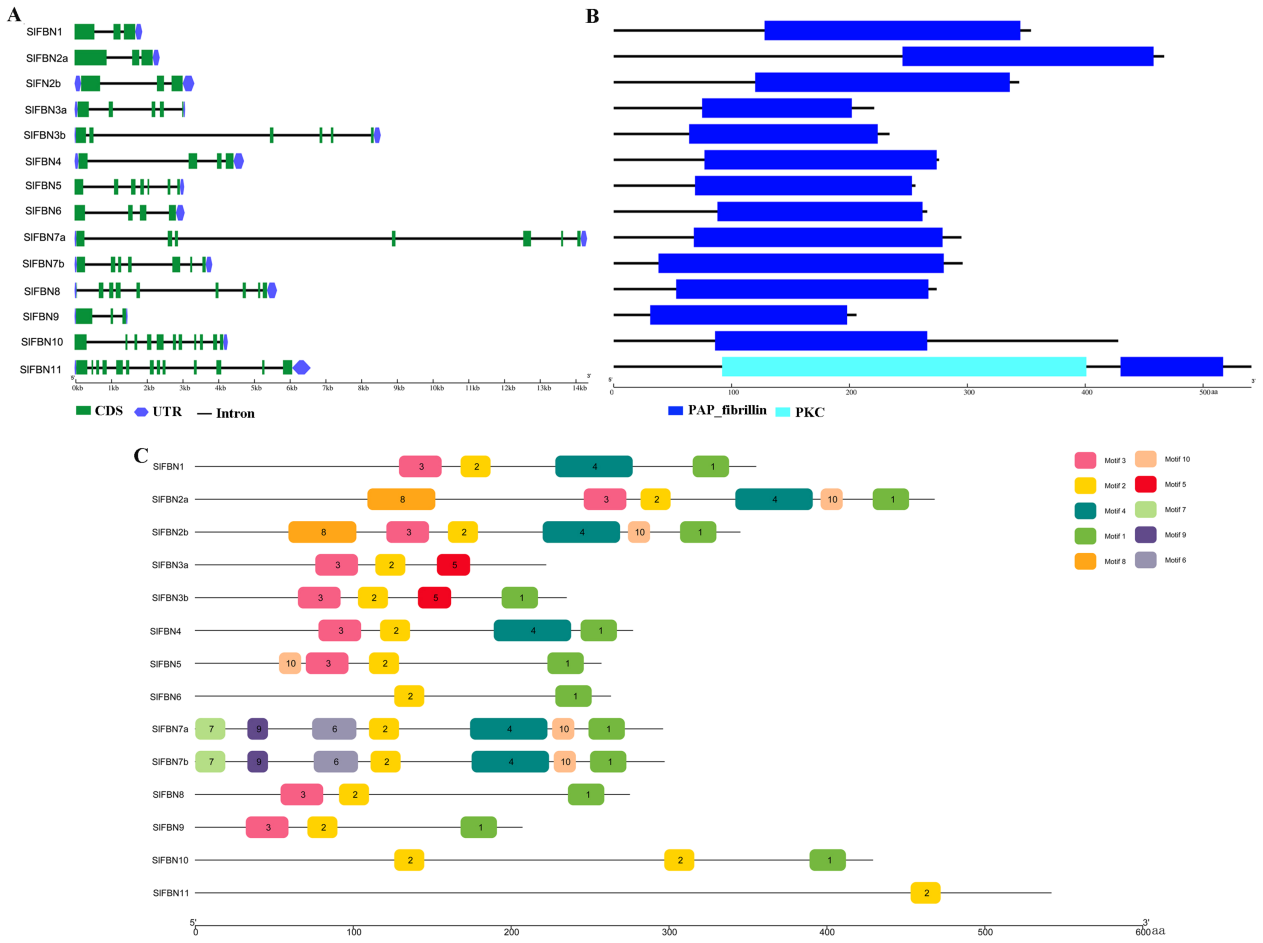
Genetic structure of *SlFBNs* (A), conserved domains (B) and conserved motifs (C) of SlFBNs.

To further explore the conservation and diversification of *SlFBNs*, the exon-intron structures and conserved motifs were analyzed. The gene structure analysis showed that the intron numbers of *SlFBNs* were various, ranging from 2 to 12 (Fig. 4A). Among them, *SlFBN1*, *SlFBN2a*, *SlFBN2b*, *SlFBN9* contain two introns, which might be due to intron loss. *SlFBN10* and *SlFBN11* contained the 10 and 12 introns, respectively, which might be related to intron increase. Similar results were also found in *FBN* family genes in rice and *Arabidopsis* (Li et al., 2020).

Analysis of the conserved domains of SlFBN family proteins revealed that all SlFBNs contained the PAP domain, which was unique to this family (Fig. 4B). SlFBN11 also contained a protein kinase C domain (PKC) besides PAP domain.

The 10 conserved motifs of SlFBN family members were analyzed to further analyze the characteristics of this family proteins. The sequences of the 10 conserved motifs were listed in Table S4. The results showed that all SlFBN proteins contained Motif2. Motif1 and Motif3 existed in most of SlFBNs, and the rest motifs existed in only individual SlFBN members (Fig. 4C). SlFBN11 only contained Motif2, which indicated that SlFBN11 might show different functions from other group members, combined with the analysis of gene structure and conserved domain.

### Cis-element analysis of *SlFBN* promoter regions

**Figure 5:**
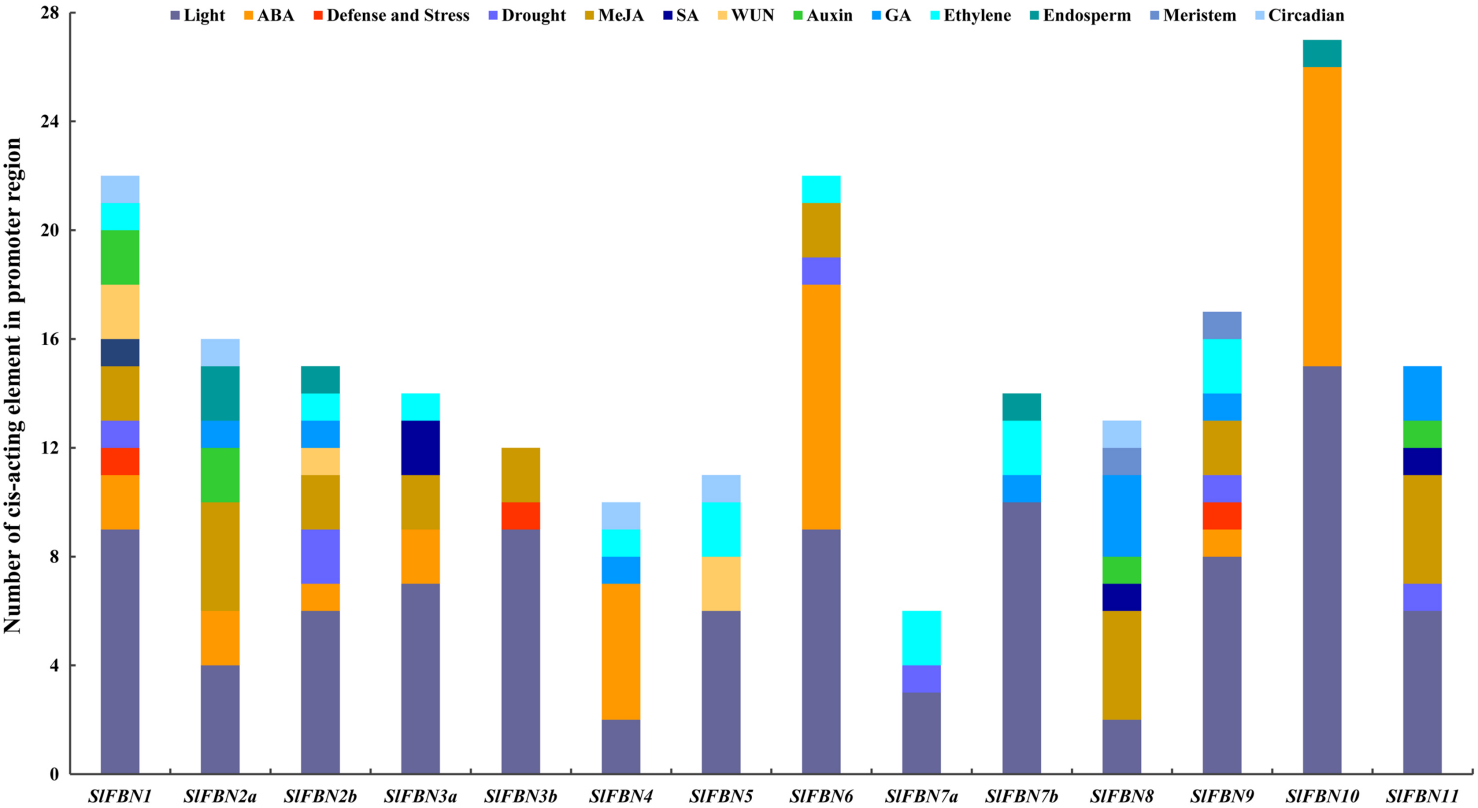
Cis-acting element distributions of *SlFBN* family gene promoters.

The cis-acting element in promoter region can partly reflect the characteristic of gene expression. The cis-acting elements in the promoter regions of *SlFBNs* were analyzed and the result showed that all of the *SlFBN* promoters contained two to 15 light response elements (Fig. 5), which indicated that the functions of *SlFBNs* might be related with light response liking photosynthesis. The hormone responsive elements, especially ethylene, MeJA and ABA, were widely distributed in the *SlFBN* promotes. The promoters of *SlFBN10*, *SlFBN6* and *SlFBN4* contained 11, nine and five ABA response elements (ABRE), respectively, suggesting that these genes might be directly or indirectly involved in ABA response pathway. In addition, auxin, gibberellin and salicylic acid response elements were distributed in some *SlFBN* promoters. Drought response element (MBS), pathogen response element (W-box), trauma response element (WUN-Motif) and defense and stress response element (TC-rich repeats) were distributed in the 6, 5, 3 and 3 *SlFBN* gene promoters, respectively. In addition to the above elements, individual *SlFBN* promoters also distributed endosperm expression elements (GCN4-Motif), meristematic expression regulation elements (CAT-box) and circadian control elements (circadian).

### Expression patterns of *SlFBNs* in different tissues

**Figure 6:**
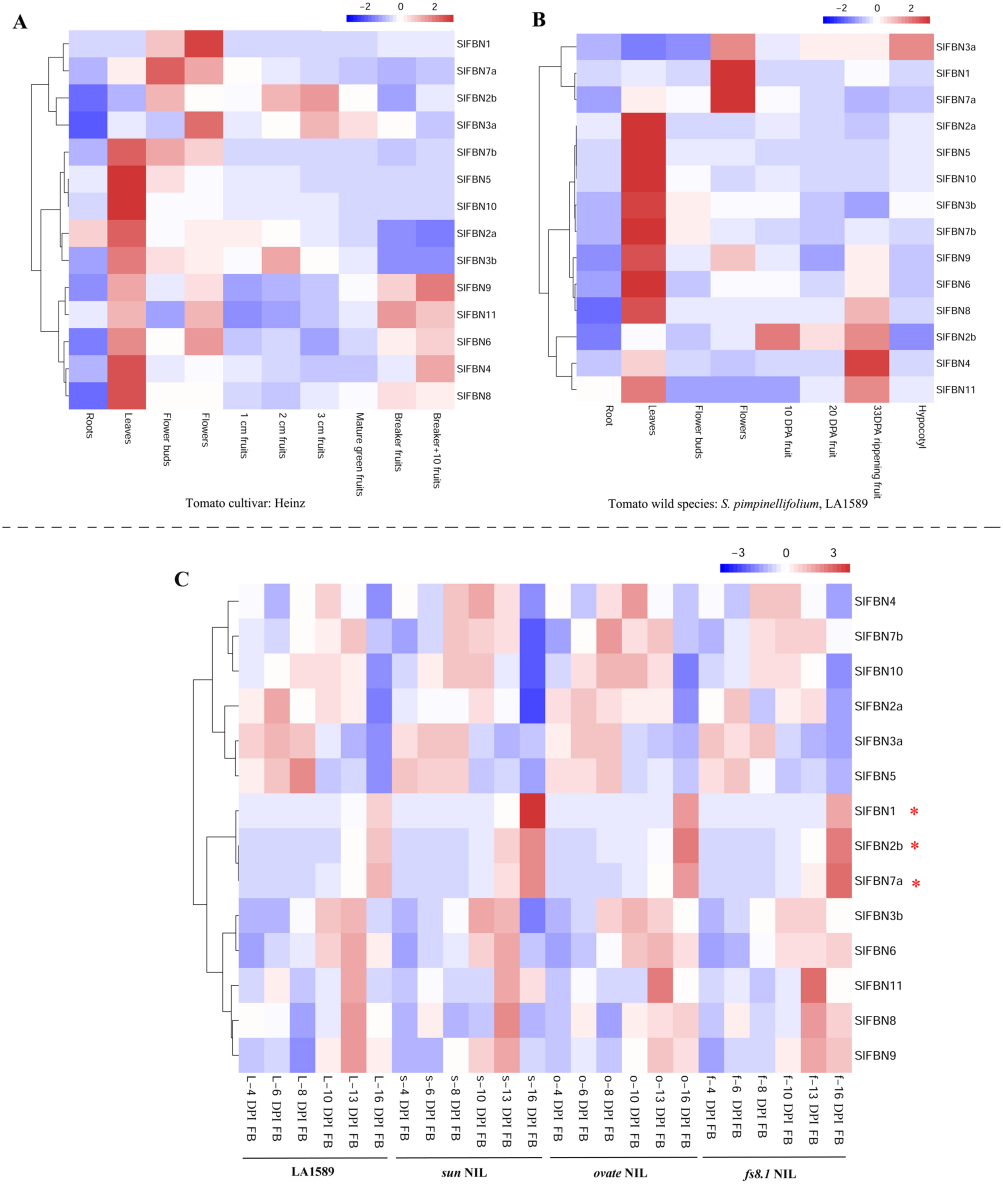
Expressions of *SlFBNs* in different organs. (A) Expression profile of *SlFBNs* in different organs of cultivated tomato cultivar Heniz; (B) expression profile of *SlFBNs* in different organs of wild species *S. pimpinellifolium* LA1589; (C) expression profile of *SlFBNs* in LA1589, *sun*, *ovate* and *fs8.1*. The three red asterisks represent the differentially expressed genes between LA1589 and the three fruit-shape NILs (*sun*, *ovate* and *fs8.1*). Scale bars in A, B and C represent log_2_-transformed FPKM values.

In order to explore the biological functions of *SlFBNs* in tomato growth and development, the expression patterns of *SlFBNs* in different tissues of tomato cultivar Heinz (Fig. 6A) and wild species *S. pimpinellifolium* LA1589 (Fig. 6B) were analyzed using published transcriptome data. Similar expression characteristics, which was that most of *SlFBNs* were preferentially expressed in leaves, were observed in Heinz and LA1589. Meanwhile, In Heinz, four *SlFBNs* (*SlFBN1*, *SlFBN2b*, *SlFBN3a* and *SlFBN7a*) were highly expressed in flowers. In LA1589, except *SlFBN2b*, the other three *SlFBNs* were also highly expressed in flowers. In addition, the expressions of some *SlFBNs* (*SlFBN4*, *SlFBN6*, *SlFBN8*, *SlFBN9* and *SlFBN11*) were increased with fruit ripening both in Heinz and LA1589, suggesting that these genes might be involved in regulating tomato fruit ripening.

The *SlFBN* expressions in flower meristems at 4, 6, 8, 10, 13 and 16 days post-initiation of floral meristem (DPI) of LA1589 and three fruit shape NILs (*sun*, *ovate* and *fs8.1*) were analyzed to further analyze their expression characteristics at different flower development stages (Fig. 6C). It was found that there was no significant difference in the expressions of 11 *SlFBNs* at different flower meristem stages. Notably, *SlFBN1*, *SlFBN7a* and *SlFBN2b* with high expressional levels in Heinz and LA1589 flower showed significantly higher expressions in three NILs than wild type at 16 DPI. The result indicated that *SlFBN1*, *SlFBN7a* and *SlFBN2b* might play important roles in tomato fruit early differentiation.

**Figure 7:**
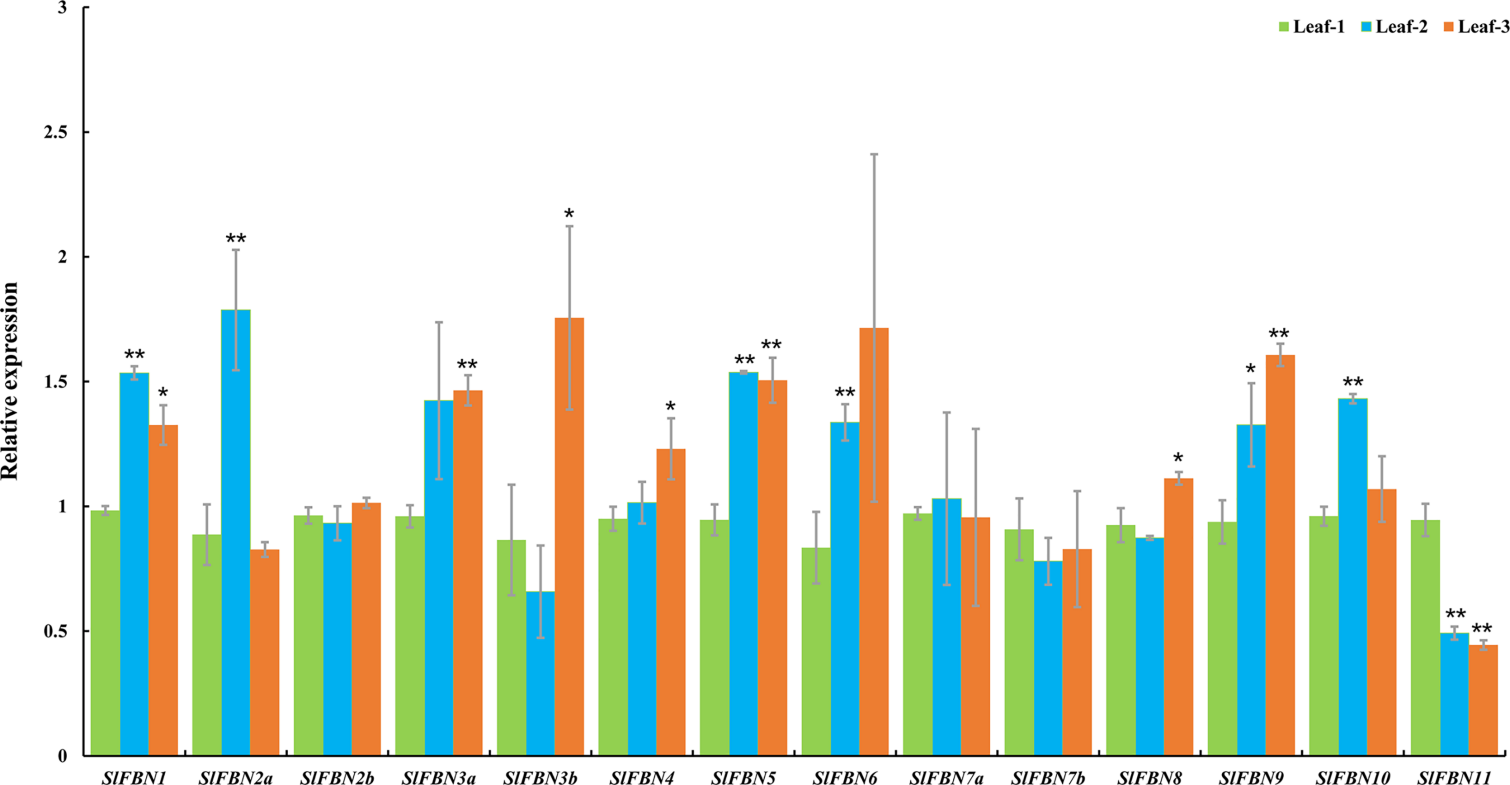
Expressions of *SlFBNs* at different development stages of tomato leaf. The expression levels of *SlFBNs* were tested by qRT-PCR and estimated by the 2^−ΔΔCT^ method. Leaf-1, Leaf-2 and Leaf-3 represent young, mature and senescent leaves, respectively; The error bars show the standard error (SE) of three biological replicates. The *p* value was calculated through *t*-test. Asterisk indicate the significant difference compared with control (Leaf-1). * and ** indicate *p* < 0.05 and *p* < 0.01, respectively.

Tissue expression analysis showed that *SlFBN* family genes were mostly expressed preferentially in leaves. The expressions of *SlFBNs* at different development stages of tomato leaf were analyzed using qRT-PCR to explore the possible functions in tomato leaf development (Fig. 7 and Table S5). The expressions of 11 *SlFBNs* (except *SlFBN2b*, *SlFBN7a* and *SlFBN7b*) in young leaves were significantly different from those in mature or senescent leaves. These *SlFBNs* showed up-regulated expressions with leaf development except *SlFBN11*, which showed opposite trend. The results indicated that *SlFBN* family genes generally perform functions during leaf development. The study of *FBN1* in bell pepper and tomato confirmed this result (Deruère et al., 1994).

### Expression profiles of *SlFBNs* under *Pst* DC3000 treatment

**Figure 8:**
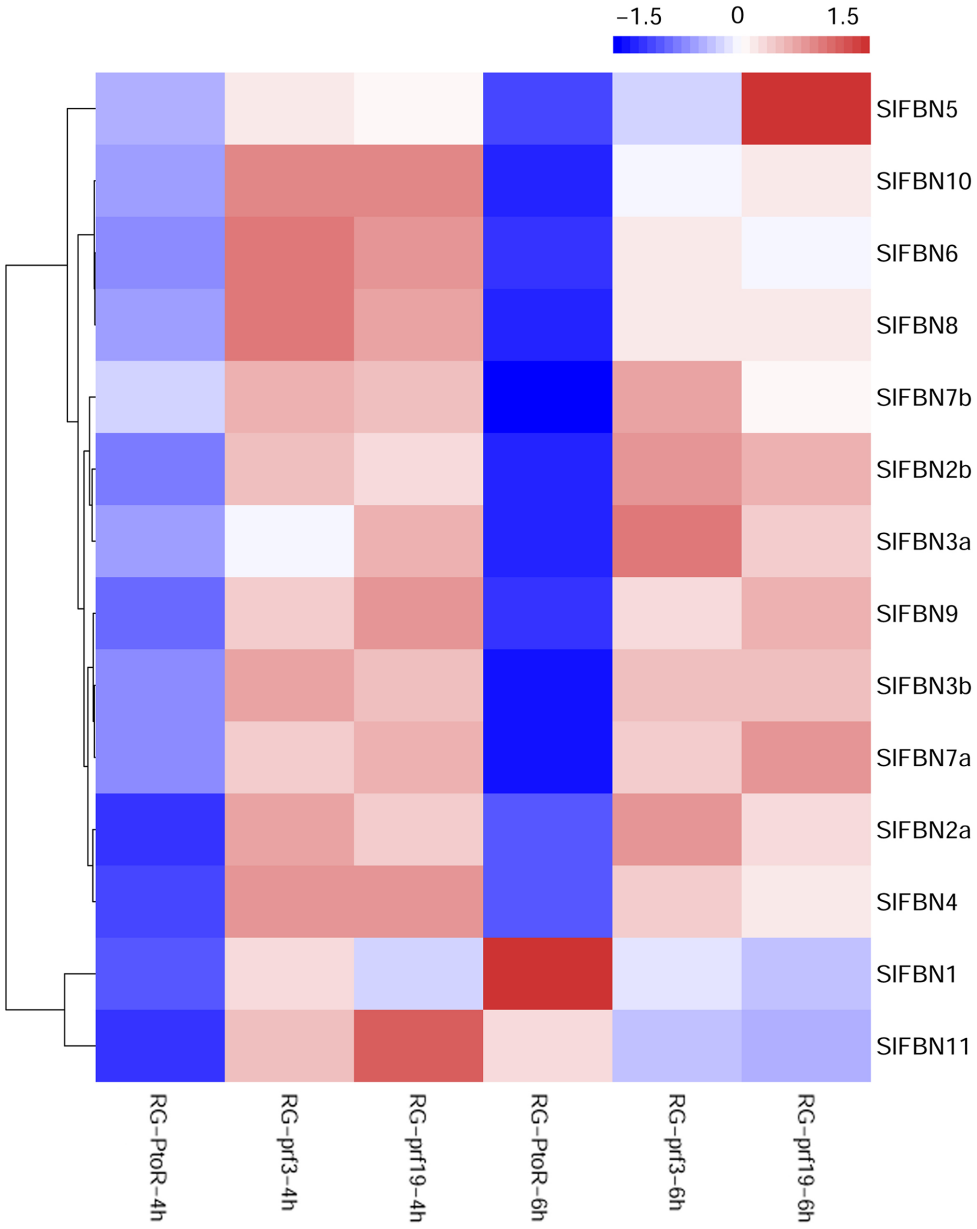
Expressions of *SlFBNs* under *Pst* DC3000 treatment. The scale bar represents log_2_-transformed FPKM values.

Previous studies showed that *FBN* family genes played important regulatory roles in stress response. The published transcriptomic data were used to analyze the expression characteristics of *SlFBN* family genes in tomato varieties with different resistances (RG-PtoR: resistant, RG-prf3: sensitive and RG-prf9: sensitive) under *Pst* DC3000 treatment (Fig. 8). The result showed that all of *SlFBN* family genes could respond to *Pst* DC3000 treatment, and 12 *SlFBNs* showed higher expression levels in resistant varieties than in sensitive varieties. The expressions of *SlFBN1* and *SlFBN11* in resistant varieties were higher than in sensitive varieties at 4 h, while the opposite trends were observed at 6 h under *Pst* DC3000 treatment.

### Expression profiles of *SlFBNs* under ABA treatment

**Figure 9:**
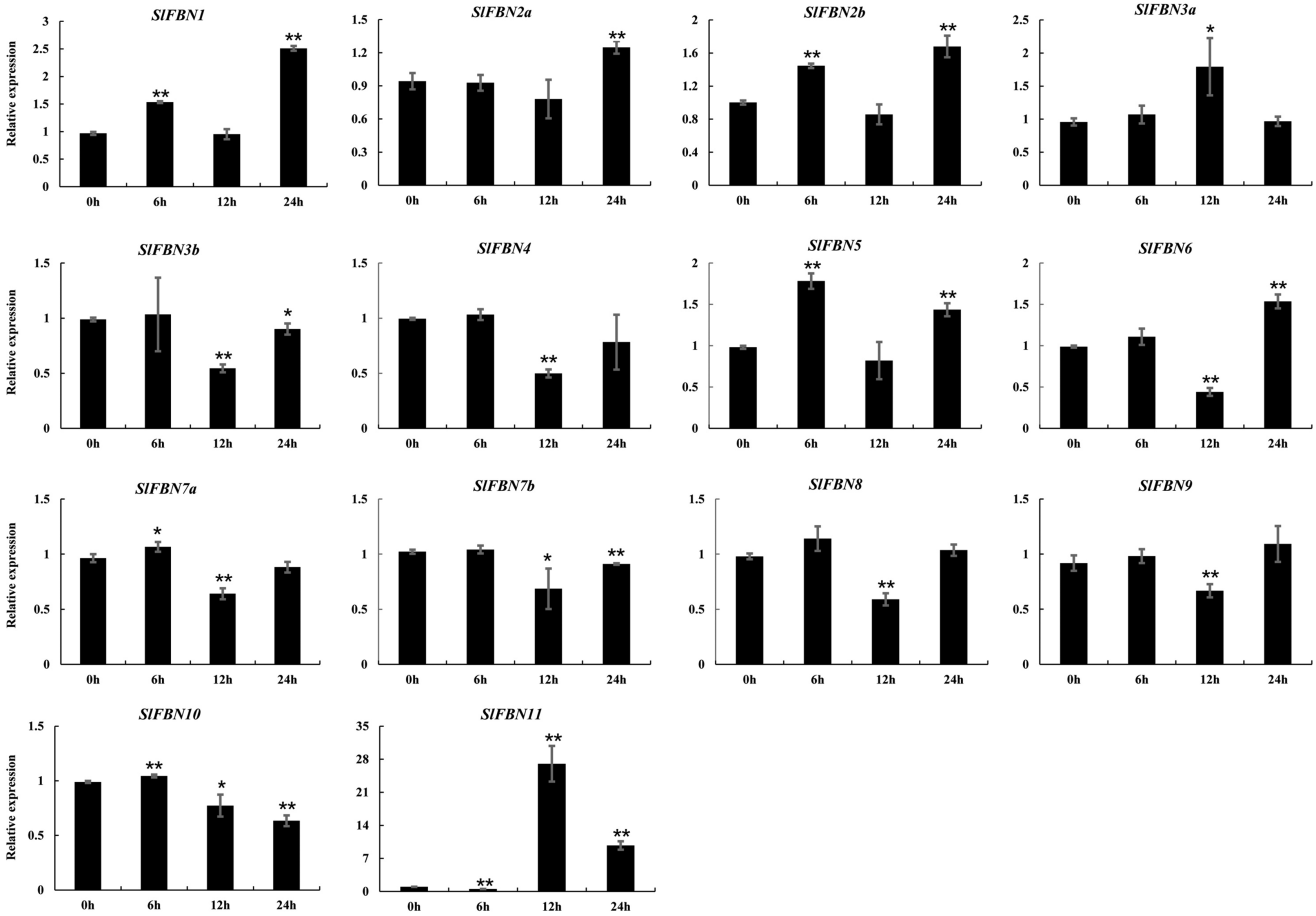
Expressions of *SlFBNs* under ABA treatment. The expression levels of *SlFBNs* were tested by qRT-PCR and estimated by the 2^−ΔΔCT^ method. 0, 6, 12 and 24 h represent 0, 6, 12 and 24 h under ABA treatment, respectively; The error bars show the standard error (SE) of three biological replicates. The *p* value was calculated through *t*-test. Asterisk indicate the significant difference compared with control (0 h). Asterisks (* and **) indicate *p* < 0.05 and *p* < 0.01, respectively.

The ABA response elements were generally distributed in *SlFBN* promoter regions. The expression levels of *SlFBNs* under ABA treatment were analyzed by qRT-PCR (Fig. 9 and Table S6). It was found that all of *SlFBNs* had significantly differences compared to the control. Compared to the 0 h, the expressions of *SlFBN1*, *SlFBN2a*, *SlFBN2b*, *SlFBN3a* and *SlFBN5* were up-regulated, and the expressions of *SlFBN3b*, *SlFBN4*, *SlFBN7b*, *SlFBN8* and *SlFBN9* were down-regulated. The expression of *SlFBN7a* and *SlFBN10* were increased firstly and then decreased. *SlFBN6* and *SlFBN11* showed early down-regulation followed by up-regulation. Notably, *SlFBN11* showed the most significant response to ABA, and its expression increased 27.0 times at 12 h and 9.7 times at 24 h compared with the 0 h, suggesting that this gene was likely to involved in the ABA signal pathway.

## Discussion

FBN family proteins were early discovered in fibrils of chromoplasts (Deruère et al., 1994) and were involved in the various plant tissues growth and development, stress and hormone signal response (Jiang et al., 2020; Liu et al., 2018). In recent years, FBN gene families have been identified in several plants, such as rice (Li et al., 2020), cucumber (Kim et al., 2018) and wheat (Jiang et al., 2020). In this study, 14 *FBN* family genes were identified in tomato genome (Fig.1 and Table S2) and unevenly distributed in the chromosomes (Fig. 2). The similar numbers of FBN family were found in diploid plants such as rice (Li et al., 2020) and *Arabidopsis* (Singh & McNellis, 2011), but less than that in heterogenous hexaploid wheat, which might be related to the genome sizes and gene duplication events. Subcellular localization prediction found that most (11/14) of *SlFBNs* were located in chloroplasts (Table S2), and similar results were found in the studies of FBN family in wheat (Jiang et al., 2020) and rice (Li et al., 2020), suggesting that *FBN* family gene might be involved in controlling chloroplast structure. Phylogenetic analysis showed that *SlFBN* family genes were divided into 11 groups (Fig. 1). *SlFBN11* belonging to group11 were significantly different from other group members in gene structure and conserved domains, suggesting that the gene might undergo new functionalization.

The gene structures of *SlFBNs* were distant with 2 to 12 introns (Fig. 3A), but the members in the same group showed similar intron numbers and the conserved motif arrangement suggesting that they might have similar functions and the members in different groups have diverse functions, especially *SlFBN11*. The concordant results were found in cucumber (Kim et al., 2018) and rice (Li et al., 2020). The collinearity analysis showed that tomato *FBN* genes have no homologous gene pairs with rice *FBN* genes and eight homologous gene pairs with *Arabidopsis FBN* genes (Fig. 3) indicating that *FBN* genes in mono- and dicotyledonous species might be divergence during evolution. We analyzed the cis-acting elements of *SlFBN* promoters to explore the *SlFBNs* expression characteristics. In *SlFBN* gene family, light and hormone response elements (such as ethylene, MeJA and ABA) are widely distributed in the promoter regions (Fig. 5), and similar regulatory elements are also widely distributed in *FBN* family genes of wheat (Jiang et al., 2020), suggesting that this family genes might be involved in the regulatory pathways of light and hormone.

The tissue expression analysis was carried out to explore the biological functions of tomato *FBN* family genes, it was found that the majority of *SlFBNs* were preferentially expressed in leaves (Figs. 6A and 6B). Further analysis of the expressions of *SlFBNs* in different development stages of leaf showed that most (11/14) of *SlFBNs* showed significantly increased expression trends with leaf maturation or aging (Fig. 7). This result was consistent with the study of subcellular localization and the GUS activity characteristics of *SlFBN1* in process of tomato leaf senescence (Georg et al., 2001), which suggested that *SlFBN1* might be involved in leaf maturation or senescence by regulating chloroplast structure. Previous studies have found that *FBN* genes were involved in regulating the fruit development of bell pepper (Kilcrease et al., 2015), satsuma mandarin (*Citrus unshiu* Marc.) (Moriguchi et al., 1998), sweet orange (*Citrus sinensis*) (Muccilli et al., 2009) and so on. In our study, *SlFBN1*, *SlFBN7a* and *SlFBN2b* were highly expressed in flowers, and the expression levels in flower meristem of three fruit shape NILs (*sun*, *ovate* and *fs8.1*) at 16 DPI were higher than those in LA1589, and the most significant differences were in *sun* (Fig. 6C). These results indicated that *SlFBN1*, *SlFBN7a* and *SlFBN2b* might be involved in regulating tomato fruit early differentiation and might be related with *sun*, *ovate* or *fs8.1* gene loci. The studies of *LeChrC* in tomato (Leitner-Dagan et al., 2006), *FBI4b* in apple and *AtFBN4* in *Arabidopsis* (Singh et al., 2010) showed that *FBN* genes could respond to pathogen infection. Through analysis of the transcriptomic data, all of *SlFBNs* could respond to *Pst* DC3000 treatment and most (12/14) of them showed higher expressions in resistant varieties than in sensitive variety (Fig. 8), which verified the previous studies. Analysis of the response of *SlFBN* family genes to ABA treatment showed that all of *FBNs* in tomato showed significant response to ABA treatment, especially *SlFBN11* (Fig. 9), suggesting that this gene might play certain roles in the ABA signal pathway. This result was different from the *FBN* family study in rice (Jiang et al., 2020) indicated that functional differentiation was occurred in *FBN* family between mono-and dicotyledons. Together, most of *SlFBN* family genes were involved in leaf development and all of them could respond to *Pst* DC3000 and ABA treatments. *SlFBN1*, *SlFBN7a* and *SlFBN2b* might play roles in regulating tomato fruit differentiation. In addition, *SlFBN11* might show different functions from other *SlFBNs*.

## Conclusions

We identified 14 *FBN* genes in tomato genome, which were divided into 11 groups and unevenly distributed on seven chromosomes. There were eight *FBN* homologous gene pairs between tomato and *Arabidopsis* and no homologous gene pairs between tomato and rice. The *FBN* gene structures were divergent. The analysis of cis-acting elements found that hormone responce elements were extensive discovered in *SlFBN* promoter regions. The results of expression analysis were found that *SlFBN* family genes might show certain functions in leaf development, fruit differentiation, stress and hormone responses. These results could provide relevant information for further study on the biological functions of *FBN* family genes.

## Supplemental Information

10.7717/peerj.13414/supp-1 Supplemental Information 1 Primers used in qRT-PCR analysis of *SlFBNs*.

10.7717/peerj.13414/supp-2 Supplemental Information 2 Physical and chemical properties of FBN family in tomato.

10.7717/peerj.13414/supp-3 Supplemental Information 3 Homologous *FBN* gene pairs between tomato and *Arabidopsis*.

10.7717/peerj.13414/supp-4 Supplemental Information 4 The 10 conserved motifs of SlFBN family proteins.

10.7717/peerj.13414/supp-5 Supplemental Information 5 Raw data of *SlFBNs* expressions at different development stages of tomato leaf for data analyses and preparation for Figure 7. The expression levels of *SlFBNs* were tested by qRT-PCR and estimated by the 2^−ΔΔCT^ method. Leaf-1, Leaf-2 and Leaf-3 represent young, mature and senescent leaves, respectively. The error bars show the standard error (SE) of three biological replicates. The *p* value was calculated through *t*-test. Asterisk indicate the significant difference compared with control (Leaf-1). * and ** indicate *p*＜0.05 and *p*＜0.01, respectively.

10.7717/peerj.13414/supp-6 Supplemental Information 6 Raw data of *SlFBNs* expressions under ABA treatment for data analyses and preparation for Figure 9. The expression levels of *SlFBNs* were tested by qRT-PCR and estimated by the 2^−ΔΔCT^ method. 0, 6, 12 and 24 h represent 0, 6, 12 and 24 h under ABA treatment, respectively. The error bars show the standard error (SE) of three biological replicates. The *p* value was calculated through *t*-test. Asterisk indicate the significant difference compared with control (0 h). * and ** indicate *p*＜0.05 and *p*＜0.01, respectively.

## Additional Information and Declarations

### Data Availability

The following information was supplied regarding data availability: The raw data are available in the Supplemental Files.
